# A survey on the state of nanosafety research in the European Union and the United States

**DOI:** 10.1007/s11051-018-4434-9

**Published:** 2018-12-20

**Authors:** Albert Duschl, Gabriele Windgasse

**Affiliations:** 1 Department of Biosciences, University of Salzburg, Hellbrunner Strasse 34, 5020 Salzburg, Austria; 2 Environmental Health Investigations Branch, California Department of Public Health, 850 Marina Bay Parkway, Richmond, CA 94804 USA

**Keywords:** Environmental health, Nanomaterials, Nanoparticles, Nanosafety, Survey, International perspective

## Abstract

Researchers in the field of environmental safety and health of nanomaterials (nanosafety) were surveyed between September 2017 and January 2018 to obtain a snapshot on the state of investigations in this field. The data received from 84 respondents give information on exposure pathways, methods, biologic and toxic effects, dose metrics and range, criteria for selecting benchmark materials, and on problems that are considered urgent in the field. The results can help guide research strategies and funding schemes related to nanosafety of nanoparticles. It is intended to repeat this survey every few years, to follow developments of the field.

**Electronic supplementary material:**

The online version of this article (10.1007/s11051-018-4434-9) contains supplementary material, which is available to authorized users.

The European Union and the USA have established a dialogue on the environmental health and safety of nanomaterials (US-EU NanoEHS, n.d.), which is pursued through annual meetings and the “Communities of Research” (CoR), in which researchers from both sides of the Atlantic jointly consider topics pertaining to nanosafety, including research needs and funding priorities. We presented a draft of the survey to the members of several CORs in September 2017 and revised the survey based on the input we received. Between September 2017 and January 2018, the CoR on human toxicity conducted a survey on the state of nanosafety research. The CoR intends to repeat this survey every 2 to 3 years, to follow developments of the field. The survey was distributed through channels that are important to this community, including the US-EU NanoEHS participant list, the EU NanoSafety Cluster (n.d.), relevant scientific meetings (e.g., EUROTOX Bratislava, APS Hatfield, BNN Graz), and professional organisations (including the Society of Toxicology’s Nanotoxicology Specialty Section). We requested that participants forward the survey to their network.

We present here some of the data and tentative conclusions derived from them. The full set of primary data is available via the US-EU NanoEHS website and the EU NanoSafety Cluster website, as well as via the supplementary data to this article.

The anonymous survey was answered by 84 persons. A larger data sample would have been desirable but considering a general “survey fatigue” among scientists, we consider that number to be sufficient to get a general idea of the present status of investigations among the participants. Questions 1–9 allowed multiple answers. The resulting percentages may add up to more than 100%.

## Academic background

The majority of respondents identified themselves as having a background in toxicology/medicine/pharmacology. Material sciences, biology, and chemistry were about equally represented. The distribution strategy aimed at experts in nanosafety and nanotoxicology, which is reflected in the responses.

## Exposure pathways

A series of questions aimed at information on what subjects are investigated and how. Publications may not fully represent ongoing scientific activities, and hard data on the focus of current research are difficult to come by. Our survey showed that more than 60% of respondents work on the inhalation pathway, which reflects safety concerns associated with this route of uptake. Ingestion and dermal uptake were in 2nd and 3rd place (30% and 23%, respectively). It is interesting that the dermal route is mentioned by nearly a quarter of respondents, even though the skin is often considered to be a rather efficient barrier. Medical conditions and consumer products may play a role here. Seven participants (8%) indicated investigating injections or intravenous exposures. Interestingly, a Google Scholar™ search (1. 11. 2018) for (nanosafety or nanotoxicology) plus one of the three exposure pathways shows a different ranking. There are 1570 articles listed for inhalation, but dermal leads ingestion by 892 articles versus 843. The attention seems to have shifted away from dermal exposure more recently, indicating that this pathway is now considered to be of lower concern than in the past. The stronger emphasis on ingestion in our survey may also be related to the fact that engineered nanomaterials were present in numerous cosmetic products for a longer time, with substantial media attention, whilst widespread use in food is a more recent development.

## Type of nanomaterials

Seventy-five percent of respondents indicated they investigate unbound/free nanomaterials, and 65% indicate they investigate aggregated/agglomerated materials. Checking multiple answers was possible and the respondents may have referred to both: the unbound discrete nanomaterials and their agglomerates/aggregates. In contrast, only 35% of researchers state they work with matrix-bound nanomaterials. This can be interpreted either by a lower concern about particles as components of a solid matrix or by more difficult methodical challenges of matrix-bound particles. Questions like this are especially relevant to pursue over time, as additional nano-enabled products reach the consumers, and product life-cycle concerns and breakdown products have to be considered. From an occupational safety and health aspect, it is important to note that 75% of the respondents indicated that they work with unbound/free materials. According to this result, measures are needed to identify exposed workers, to monitor, control, and reduce workplace exposures, and to medically monitor exposed workers. This also requires the development of occupational exposure standards and protocols for exposure and medical monitoring.

## Investigative methods

Tests performed are mainly chemical/physical characterisation or testing in vitro/in vivo. Despite high hopes for in silico methods, only 12% of respondents performed them. Given strong efforts in that direction, that number should increase in the future. Five participants indicated they were engaged in epidemiologic studies, a number that may also increase in the future.

## Biologic/toxic effects under investigation (Fig. 1)

Among biological effects studied, reactive oxygen species (ROS) take first place (66%) followed by immune system effects (40%). This strong interest in cell stress and immunity may have two reasons: first, the cell stress programme and the inflammatory programme are quick and rather unspecific responses to stimuli that challenge the integrity of the body. Observing associated endpoints, therefore, gives quick and relevant insights into biological responses, contributing to the popularity of methods aiming at these effects. Second, regulation of the inflammatory status, which includes ROS production, is associated with a large number of disease states, as cause, as contributor, or in a therapeutic role. Nanosafety and nanomedicine are equally interested in better understanding and control of these processes. Studies of neurologic effects, reproductive/developmental effects, and carcinogenesis made up 25%, 25%, and 22%, respectively. Five respondents (6%) indicated they work on cardiovascular effects.

**Fig. 1:**
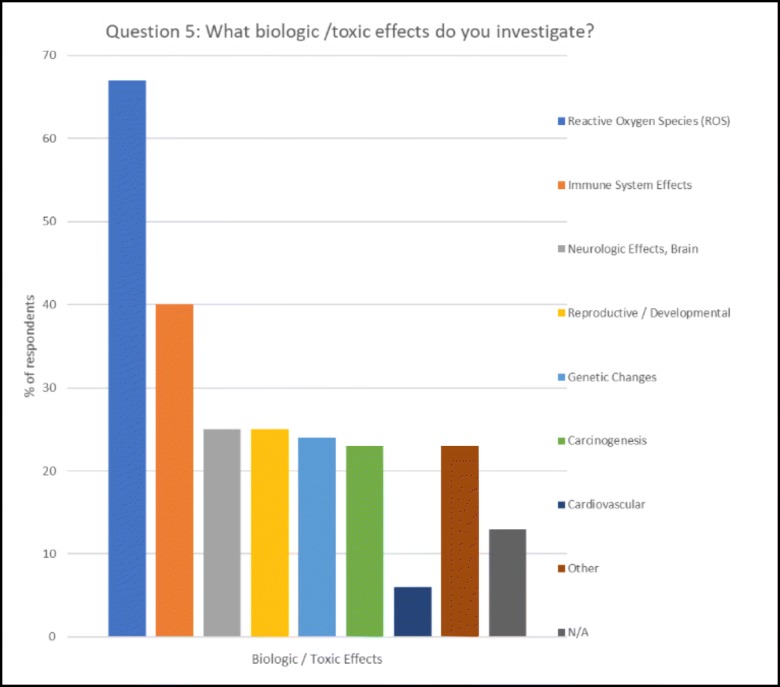
Biologic/toxic effects under investigation

Again, a Google Scholar™ search (1. 11. 2018) for (nanosafety or nanotoxicology) plus phrases that indicate effects gives a somewhat different picture. The number of listed articles is as follows: reactive oxygen species or ROS, 1180; immune, 1090; genetic or DNA, 1170; neurological or neurologic or brain, 165; reproduction or development, 916; and cancer or carcinogenesis, 832.

Reactive oxygen species are strongly linked to immune responses, so the high number of papers on both is not surprising. The lower number of respondents indicating developmental or reproductive effects may indicate that environmental toxicologists may have been underrepresented in the group of respondents since reproductive effects are mainly studied in multi-generation studies on environmental organisms. The high number of publication on cancer and genetic effects—also strongly linked—is likely due to the fact that many of them come from the medical community.

## Nanomaterial applications

The nanomaterials under investigation are associated with many potential commercial applications: paint and coatings are mentioned most frequently (51%), followed by pharmaceuticals/medicine (45%), food/food contact materials (42%), and textiles and composite materials (40% each). As the uses of nanomaterials evolve and mature, we expect to see a change in the type of applications under investigation.

## Dose metrics

One difficulty in dealing with literature is that dose/concentration is reported in different ways. The survey showed no emerging consensus. Weight in milligramme per kilogramme body weight, per millilitre blood, or per cubic metre air are all used, as are number of particles per cubic metre and surface area. At the nanoscale, the mass of the particles is less relevant than the accessibility of the particle surface to react with their environment (reactivity and/or surface area). Thirteen participants (15%) indicated they include surface area in their reporting of dose or concentration, but no participant used reactivity-based measures.

## Dose range

Doses used in experiments are to equal extents determined by reported concentrations in products or environment and by doses necessary to observe effects. ADME (adsorption, distribution, metabolism, excretion) models play a minor role. For finding a “threshold”, 46% of respondents use NOAEL and 31% use LOAEL, whilst 37% do not attempt to find a threshold dose.

## Criteria for benchmark materials (Fig. 2)

An important motivation for performing the survey was to find candidates for “benchmark materials”. Such materials can be defined as standards to which other materials can be compared to or judged. For example, what is a “typical fibre”, to which other fibres can be reasonably compared? We asked for feedback on specific criteria that would be important for defining benchmark materials. No consensus emerged. The two criteria most often mentioned as “very important” were production volume (37%) and exposure to consumers (29%). Specific descriptors of “benchmark materials” remain elusive.

**Fig. 2:**
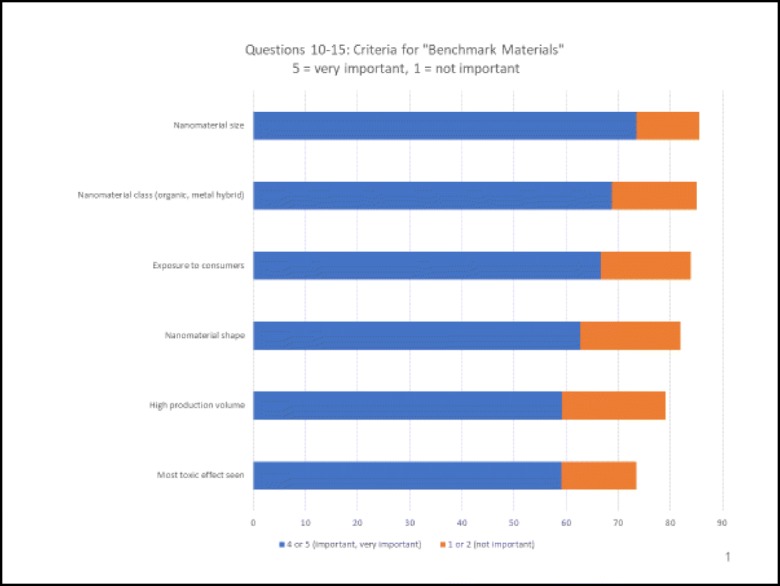
Criteria for “Benchmark Materials”

## Urgency for investigation (Fig. 3)

A list of 19 topics was provided and it was requested to rate the urgency to address them. All of them were considered very or somewhat urgent by a majority of respondents, which suggests that there is no perception of one single overriding issue in environmental safety and health of nanomaterials. However, a consensus emerged for issues rated “very urgent” by the majority of respondents: standard methods for toxicity testing (71%), worker protection (64%), biomarkers of effect (59%), and biomarkers of exposure (57%). This reflects a need for improved standardisation to allow easier data sharing and comparison, and the needs to evaluate current exposures and effects, especially in occupational safety and health. Future surveys should reveal whether the scientific community gets closer to this goal. It is planned to open a new edition of the survey in 2019 or 2020.

**Fig. 3:**
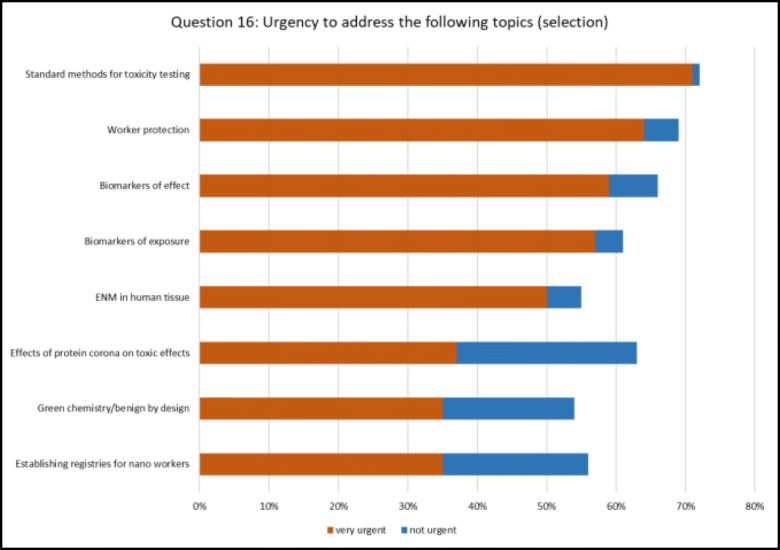
Urgency to address topics

## Electronic supplementary material

ESM 1 (PDF 219 kb)

## Abbreviations

ADME: adsorption, distribution, metabolism, excretion
CoR: community of research
LOAEL: lowest-observed-adverse-effect level
NOAEL: no-observed-adverse-effect level
ROS: reactive oxygen species

## References

[CR1] EU Nanosafety Cluster (n.d.) https://www.nanosafetycluster.eu/

[CR2] US-EU dialogue, bridging nanoEHS (n.d.) research: https://us-eu.org/

