# Supplementary material for: A survey on the state of nanosafety research in the European Union and the United States
Source: J Nanopart Res. 2018 Dec 20;20(12):335. doi: 10.1007/s11051-018-4434-9 (PMC6302049; doi:10.1007/s11051-018-4434-9)
Supplement: Supplementary file 1 — (PDF 219 kb) [file 11051_2018_4434_MOESM1_ESM.pdf]

Q1 Your academic background

Answered: 82 Skipped: 2

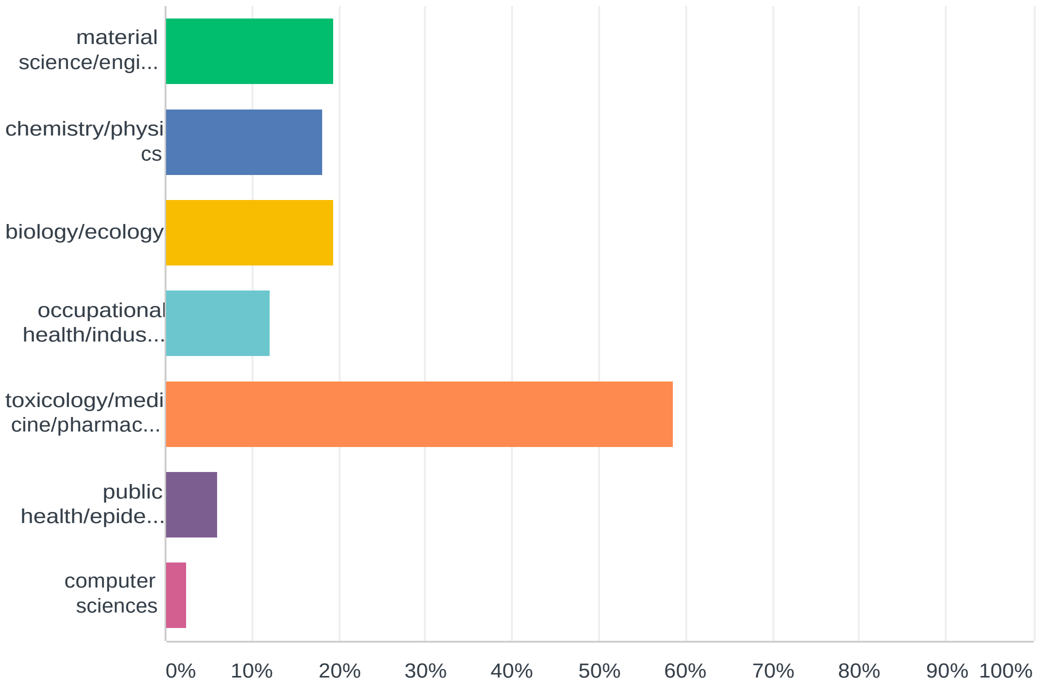

ANSWER CHOICES	RESPONSES	
material science/engineering	19.51%	16
chemistry/physics	18.29%	15
biology/ecology	19.51%	16
occupational health/industrial hygiene	12.20%	10
toxicology/medicine/pharmacology	58.54%	48
public health/epidemiology	6.10%	5
computer sciences	2.44%	2
Total Respondents: 82		

#	OTHER (SPECIFY)	DATE
1	Environmental Engineering	
2	risk analysis/environ engineering	
3	Biochemistry	
4	physiology	
5	biochemistry	

Q2 Your investigations involve the following exposure pathways:

Answered: 84 Skipped: 0

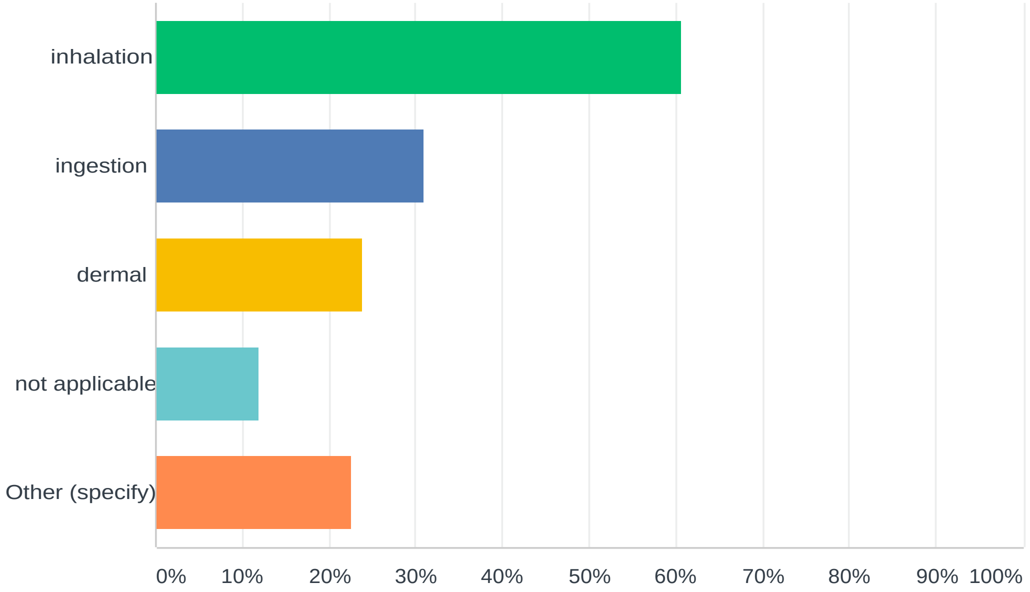

ANSWER CHOICES	RESPONSES	
inhalation	60.71%	51
ingestion	30.95%	26
dermal	23.81%	20
not applicable	11.90%	10
Other (specify)	22.62%	19
Total Respondents: 84		

#	OTHER (SPECIFY)	DATE
1	systems toxicology	
2	injection	
3	bacteria and aquatic organisms for environmentally relevant exposures, impacts, and applications	
4	in vitro	
5	Developmental exposures	
6	intravenous	
7	in vitro mammalian assays, aqueous exposure to environmental organisms, microbial cells	
8	wastewater treatment	
9	freshwater systems	
10	systemic	
11	intracoronary	
12	injection	
13	parenteral	

14	iv
15	in vitro
16	Intravenous
17	In Vitro
18	intravenous
19	blood contacting

Q3 Types of nanomaterials under investigation

Answered: 84 Skipped: 0

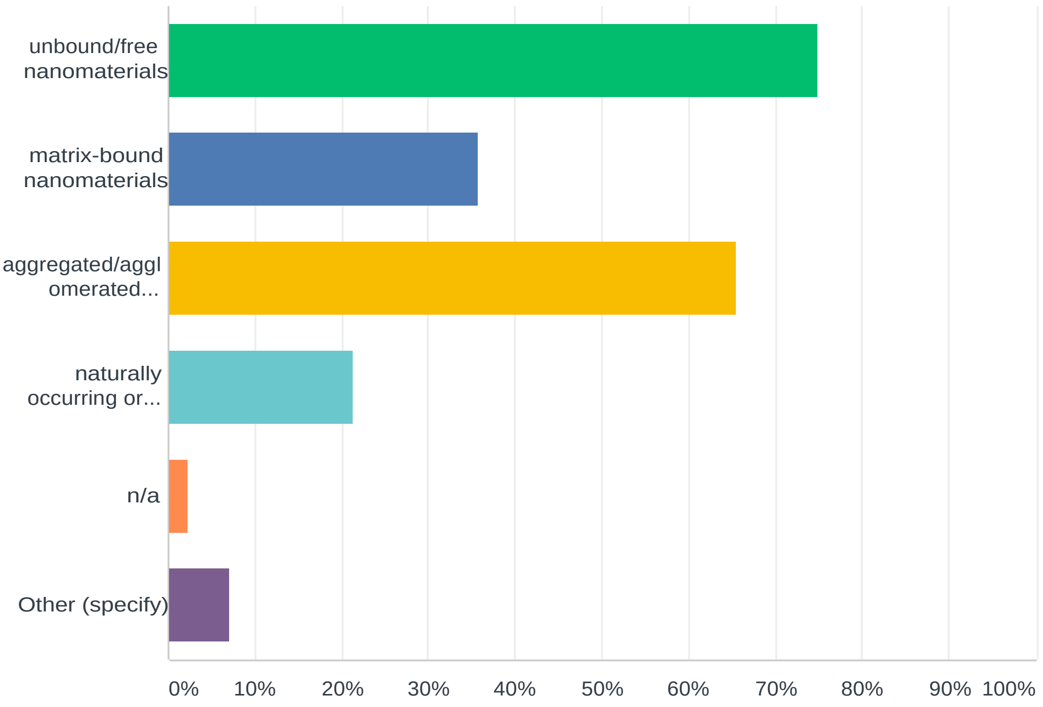

ANSWER CHOICES	RESPONSES	
unbound/free nanomaterials	75.00%	63
matrix-bound nanomaterials	35.71%	30
aggregated/agglomerated nanomaterials	65.48%	55
naturally occurring or incidental nanomaterials	21.43%	18
n/a	2.38%	2
Other (specify)	7.14%	6
Total Respondents: 84		

#	OTHER (SPECIFY)	DATE
1	reusing (open) data	
2	Metals nanoparticles	
3	engineered nanoparticles	
4	environmental transformation products	
5	Carbon nanomaterials	
6	nanomaterials in solution	

Q4 What investigative methods do you use?

Answered: 84 Skipped: 0

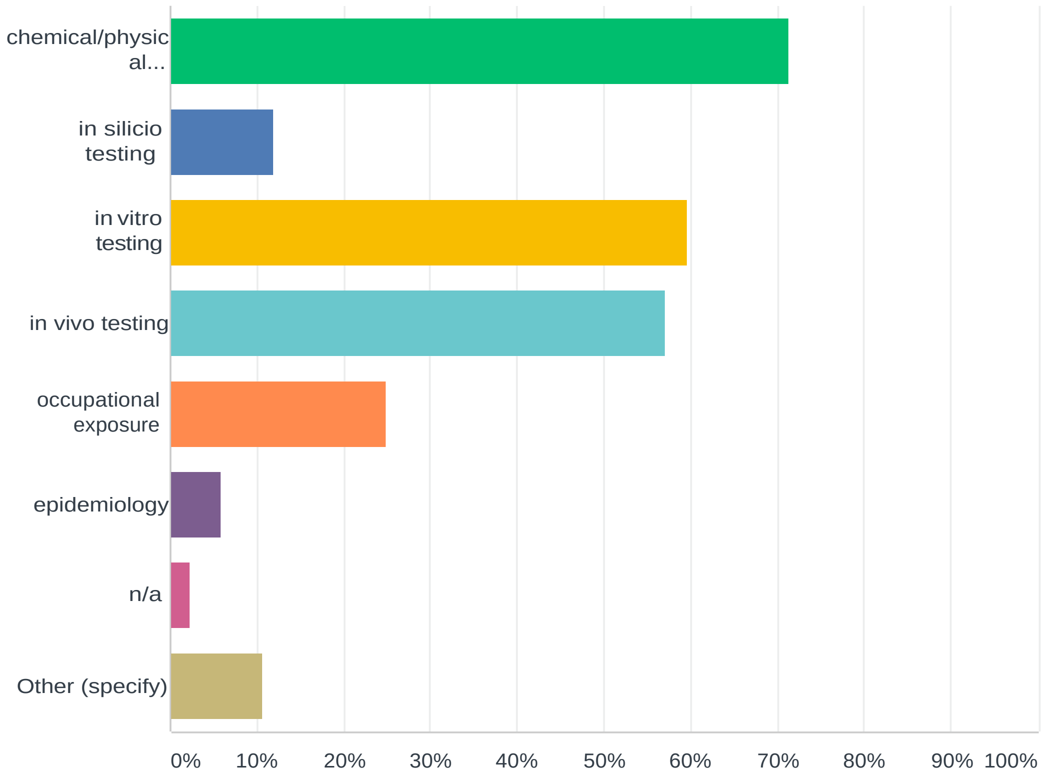

ANSWER CHOICES	RESPONSES	
chemical/physical characterization	71.43%	60
in silicio testing	11.90%	10
in vitro testing	59.52%	50
in vivo testing	57.14%	48
occupational exposure	25.00%	21
epidemiology	5.95%	5
n/a	2.38%	2
Other (specify)	10.71%	9
Total Respondents: 84		

#	OTHER (SPECIFY)	DATE
1	data integration	
2	Simulation of product use	
3	risk analysis model	
4	literature review	
5	mesocosms	
6	clinical toxicology	

7	Zebrafish
8	review for regulatory acceptance; protocol development
9	risk assessment

Q5 What biologic/toxic effects do you investigate?

Answered: 84 Skipped: 0

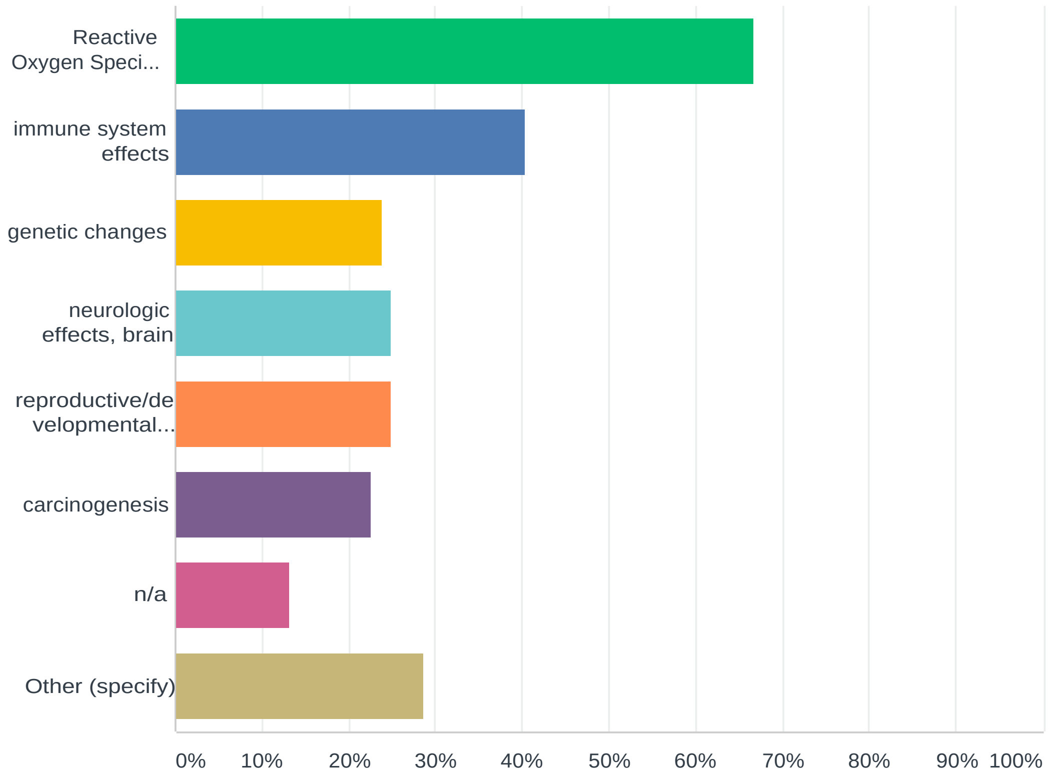

ANSWER CHOICES	RESPONSES	
Reactive Oxygen Species (ROS)	66.67%	56
immune system effects	40.48%	34
genetic changes	23.81%	20
neurologic effects, brain	25.00%	21
reproductive/developmental effects	25.00%	21
carcinogenesis	22.62%	19
n/a	13.10%	11
Other (specify)	28.57%	24
Total Respondents: 84		

#	OTHER (SPECIFY)	DATE
1	any effect is relevant	
2	cell death (bacteria), organism death (e.g., embryonic zebrafish), biomolecule reactions (e.g. glutathione)	
3	Systemic	
4	cell growth and NP uptake	
5	effects to microbial communities	

6	NP toxic effects in fluvial biofilms at molecular, functional and structural level
7	digestive impairment
8	effects to function of engineered systems
9	physiological responses
10	cardiovascular
11	histopathological changes
12	cardiovascular
13	Developmental morphology and Molecular responses
14	lung diseases
15	pharmacokinetics
16	Microbiome interactions
17	Regulatory requirements
18	cardiovascular
19	cardiovascular effects
20	systemic effects
21	Fibrosis
22	inflammation, ADME
23	cardiovascular
24	vascular

Q6 What are the potential applications for the nanomaterials you are investigating?

Answered: 84 Skipped: 0

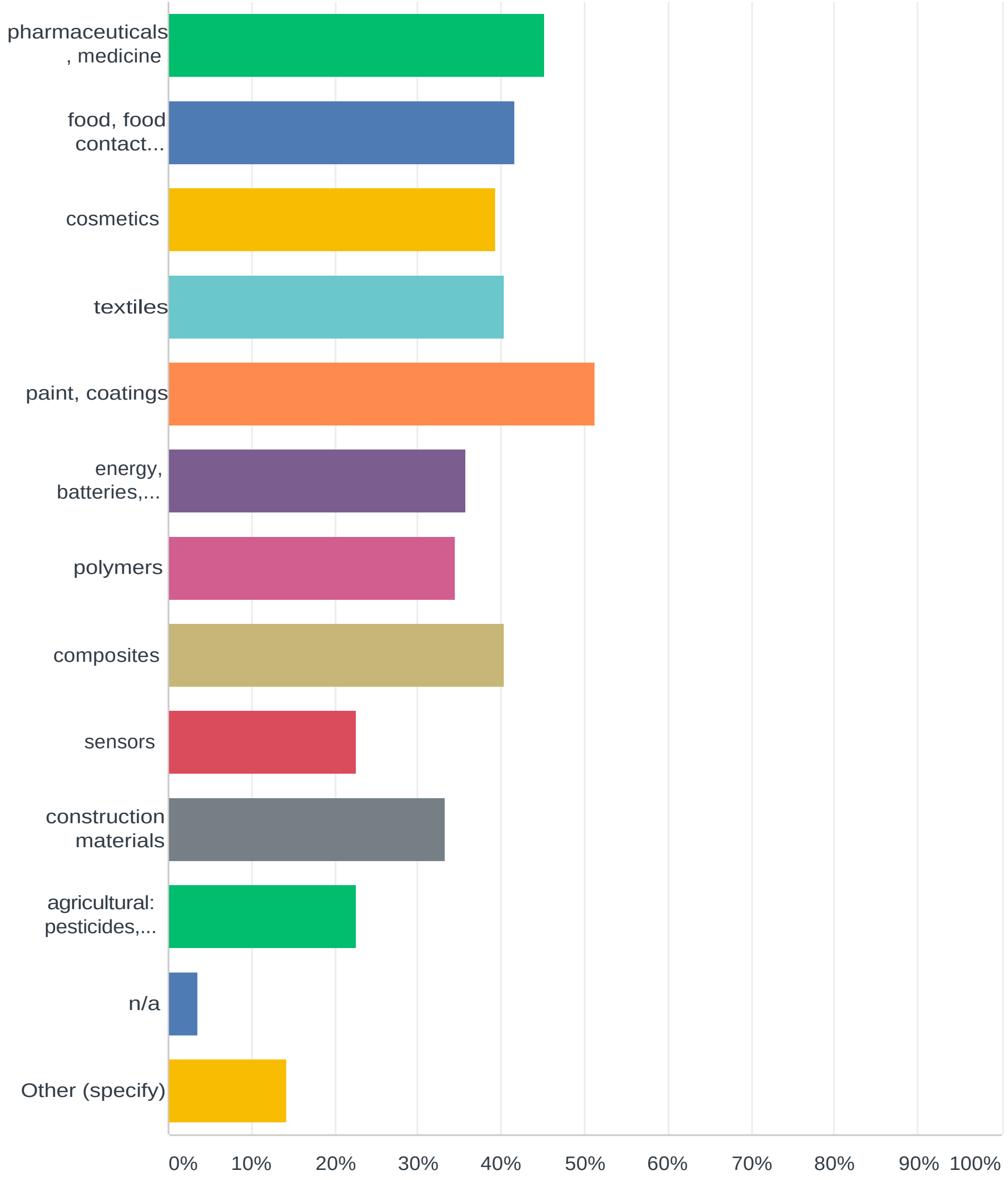

ANSWER CHOICES	RESPONSES	
pharmaceuticals, medicine	45.24%	38
food, food contact materials	41.67%	35
cosmetics	39.29%	33
textiles	40.48%	34
paint, coatings	51.19%	43

energy, batteries, electronics	35.71%	30
polymers	34.52%	29
composites	40.48%	34
sensors	22.62%	19
construction materials	33.33%	28
agricultural: pesticides, soil amendment	22.62%	19
n/a	3.57%	3
Other (specify)	14.29%	12
Total Respondents: 84		

#	OTHER (SPECIFY)	DATE
1	water	
2	water treatment	
3	Water treatment	
4	Light	
5	water treatment	
6	nano in waste stream	
7	environmental waste (waste water treatment plant effluents)	
8	newly developed nanomaterials	
9	alloys	
10	Petroleum additives	
11	medical devices	
12	construction material, aerospace/automobile composite	

Q7 How do you report dose/concentration in your experiments?

Answered: 84 Skipped: 0

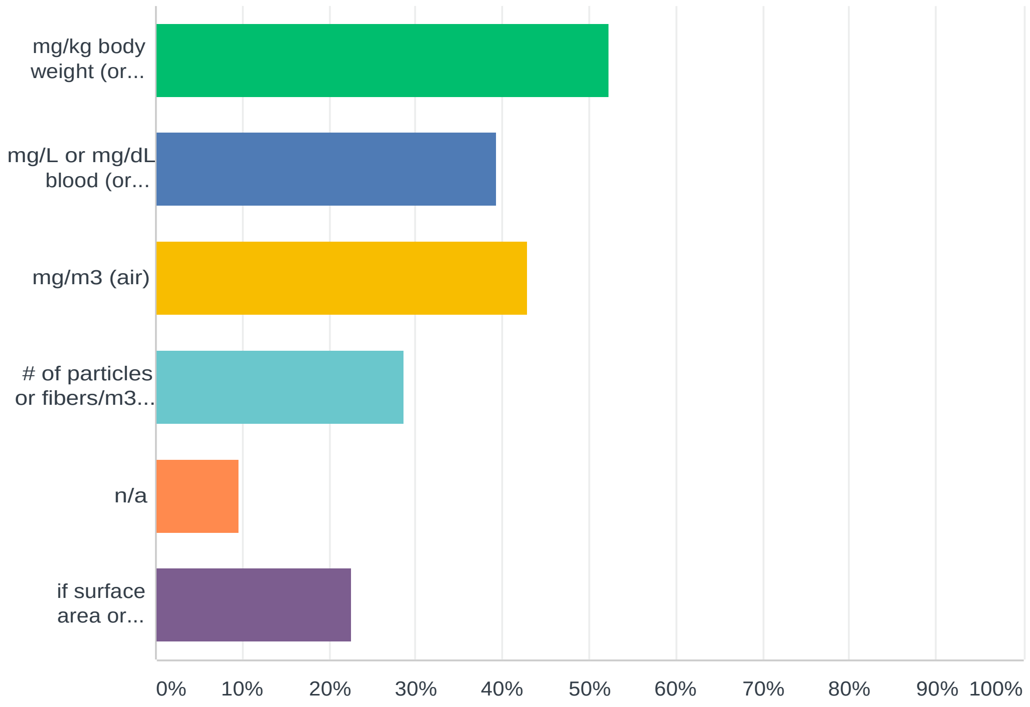

ANSWER CHOICES	RESPONSES	
mg/kg body weight (or similar)	52.38%	44
mg/L or mg/dL blood (or similar)	39.29%	33
mg/m3 (air)	42.86%	36
# of particles or fibers/m3 (air)	28.57%	24
n/a	9.52%	8
if surface area or reactivity or other, specify:	22.62%	19
Total Respondents: 84		

#	IF SURFACE AREA OR REACTIVITY OR OTHER, SPECIFY:	DATE
1	we use what is appropriate for the material	
2	surface area	
3	Particle size	
4	surface area	
5	Surface area	
6	particles/volume	
7	surface area, lung deposited surface area, attempts on reactivity, but w/o success at this point in time	
8	ug/L	
9	mg/cm2 for in vitro (or #/cm2)	

10	surface area
11	mg/m2
12	ug/ml in vitro, modeled deposition to cell layer
13	Surface area and particle number
14	surface area
15	many dose metrics evaluated
16	sa
17	surface area
18	in vitro dose is microgram per cm2
19	ppb

Q8 How do you determine the range for dose or concentration inyour toxicity experiments?

Answered: 84 Skipped: 0

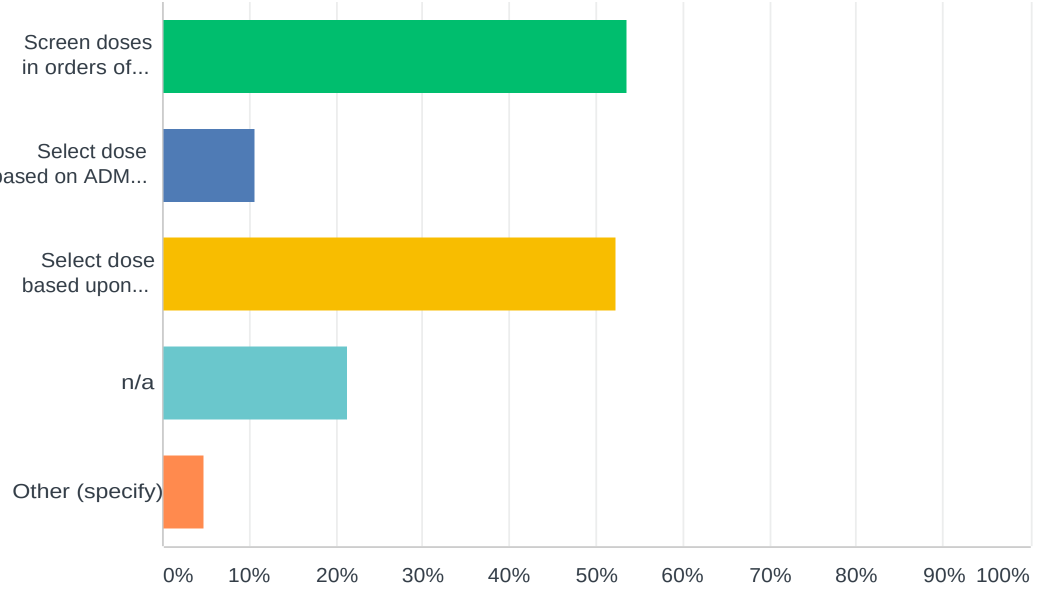

ANSWER CHOICES	RESPONSES	
Screen doses in orders of magnitude until effects are seen	53.57%	45
Select dose based on ADME (adsorption, distribution, metabolism, excretion) models for organs from which the cell line is derived	10.71%	9
Select dose based upon reported concentrations in products or environmental media	52.38%	44
n/a	21.43%	18
Other (specify)	4.76%	4
Total Respondents: 84		

#	OTHER (SPECIFY)	DATE
1	Relevant doses/exposure concentrations considering conversion of existing OELs as a starting point	
2	equivalent human dosage related to occupational exposures	
3	Combination of literature review, followed by concentration responses to optimize the ranges used	
4	occupational exposure levels or levels reported in the literature for reference materials	

Q9 Are you trying to find a "threshold" dose or concentration?

Answered: 83   Skipped: 1

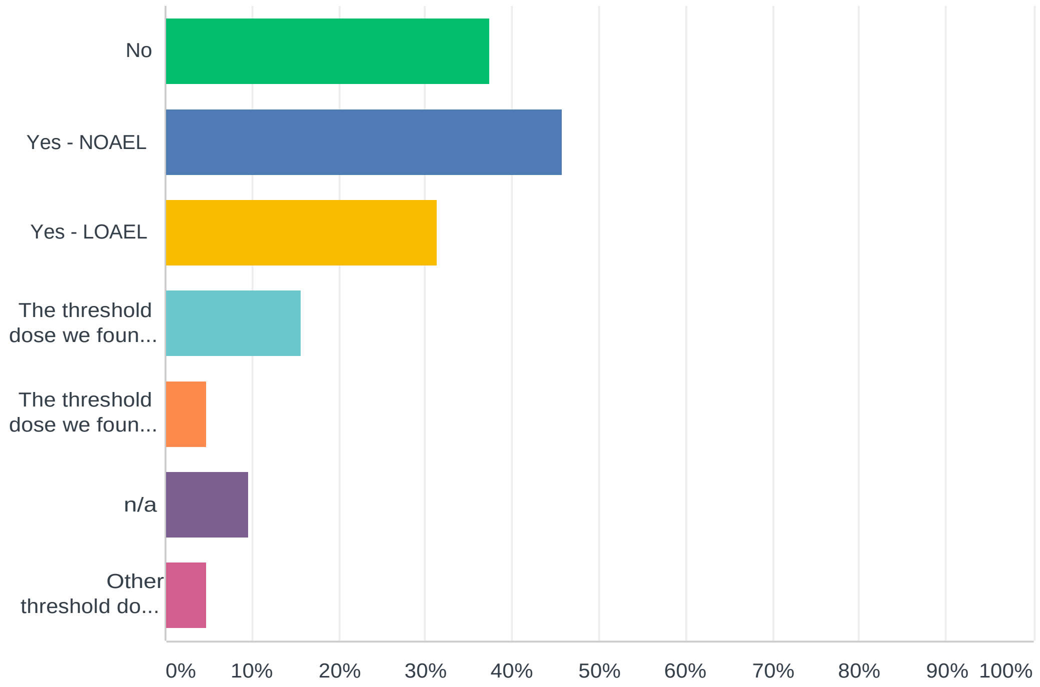

ANSWER CHOICES	RESPONSES	
No	37.35%	31
Yes - NOAEL	45.78%	38
Yes - LOAEL	31.33%	26
The threshold dose we found is different from the bulk material	15.66%	13
The threshold dose we found is similar to the bulk material	4.82%	4
n/a	9.64%	8
Other threshold dose or concentration:	4.82%	4
Total Respondents: 83		

#	OTHER THRESHOLD DOSE OR CONCENTRATION:	DATE
1	sometimes; when the focus is on the material, comparative magnitude impact for same dose is used	
2	BMD/BMDL	
3	Depends on the study	
4	Bulk concentration vs % nanoparties in airborne material	

Q10 "Benchmark materials" are defined here as a standard by which other materials can be compared or judged. To help identify "benchmark materials" for specific classes of nanomaterials, please rate the following criteria:high production volume

Answered: 81 Skipped: 3

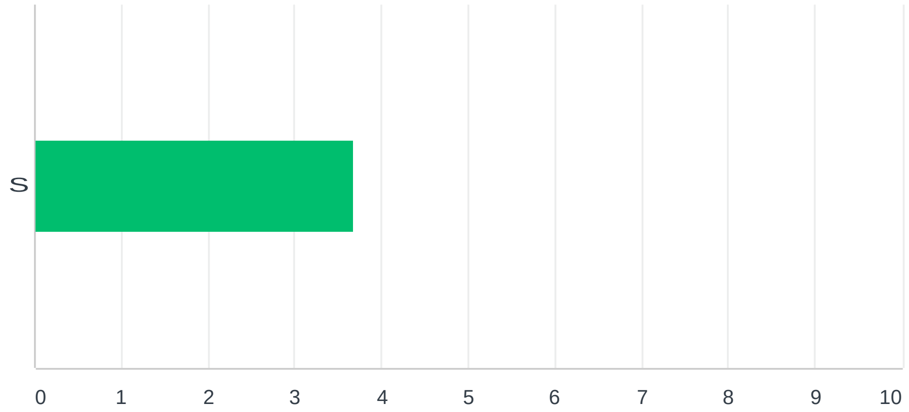

	NOT IMPORTANT	(NO LABEL)	(NO LABEL)	(NO LABEL)	VERY IMPORTANT	N/A	TOTAL	WEIGHTED AVERAGE
S	11.11%<br>9	8.64%<br>7	16.05%<br>13	22.22%<br>18	37.04%<br>30	4.94%<br>4	81	3.69

Q11 nanomaterial size

Answered: 83 Skipped: 1

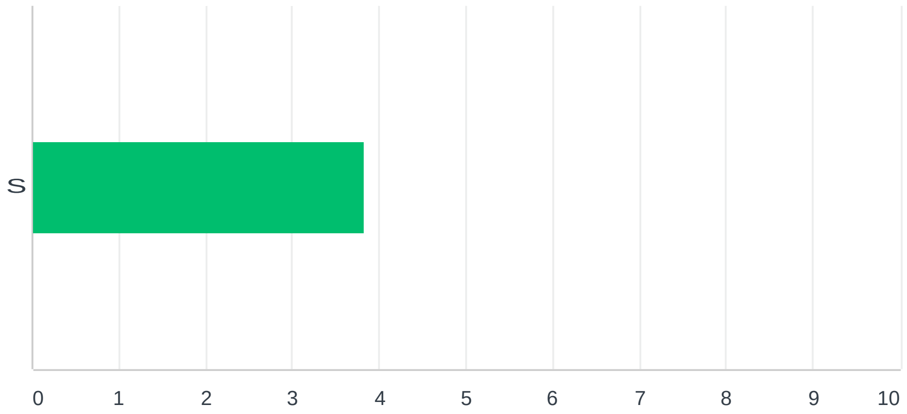

	1	2	3	4	5	TOTAL	WEIGHTED AVERAGE
S	2.41%<br>2	9.64%<br>8	14.46%<br>12	49.40%<br>41	24.10%<br>20	83	3.83

Q12 nanomaterial shape

Answered: 83 Skipped: 1

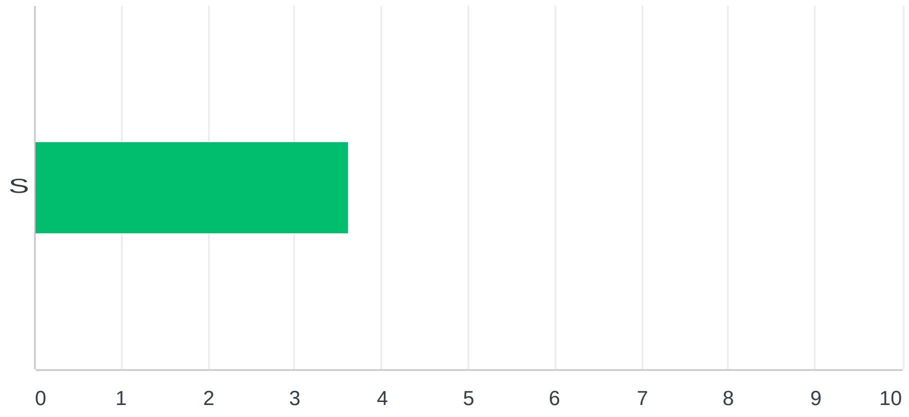

	1	2	3	4	5	TOTAL	WEIGHTED AVERAGE
S	3.61%<br>3	15.66%<br>13	18.07%<br>15	40.96%<br>34	21.69%<br>18	83	3.61

Q13 nanomaterial class (organic, metal, hybrid)

Answered: 80 Skipped: 4

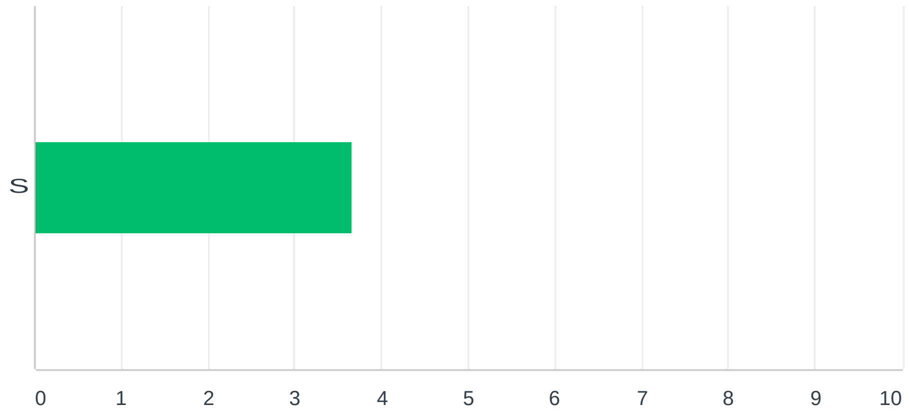

	1	2	3	4	5	TOTAL	WEIGHTED AVERAGE
S	7.50%<br>6	8.75%<br>7	15.00%<br>12	46.25%<br>37	22.50%<br>18	80	3.67

Q14 exposure to consumers

Answered: 81 Skipped: 3

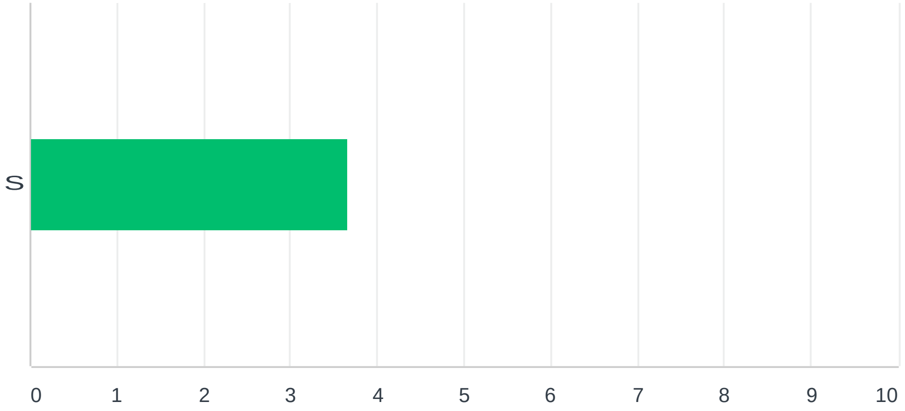

	1	2	3	4	5	TOTAL	WEIGHTED AVERAGE
S	12.35%<br>10	4.94%<br>4	16.05%<br>13	37.04%<br>30	29.63%<br>24	81	3.67

Q15 most toxic effect seen

Answered: 83 Skipped: 1

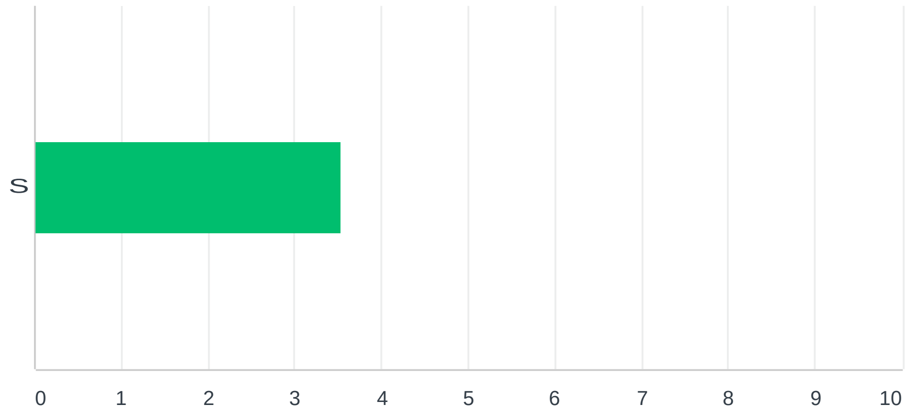

	1	2	3	4	5	TOTAL	WEIGHTED AVERAGE
S	8.43%<br>7	6.02%<br>5	26.51%<br>22	40.96%<br>34	18.07%<br>15	83	3.54

#	OTHER CRITERIA:	DATE
1	bulk counterpart, if available	
2	Surface chemistry, agglomeration state	
3	It is important that it is a well-known material that all can relate to	
4	Dissolution rates	
5	stability over time including during storage	
6	commercial product that has consistent physical and chemical characteristics across different production lots	
7	Amount of published literature; availability	
8	Significant toxicology database	

Q16 Urgency to address the following topics:

Answered: 84 Skipped: 0

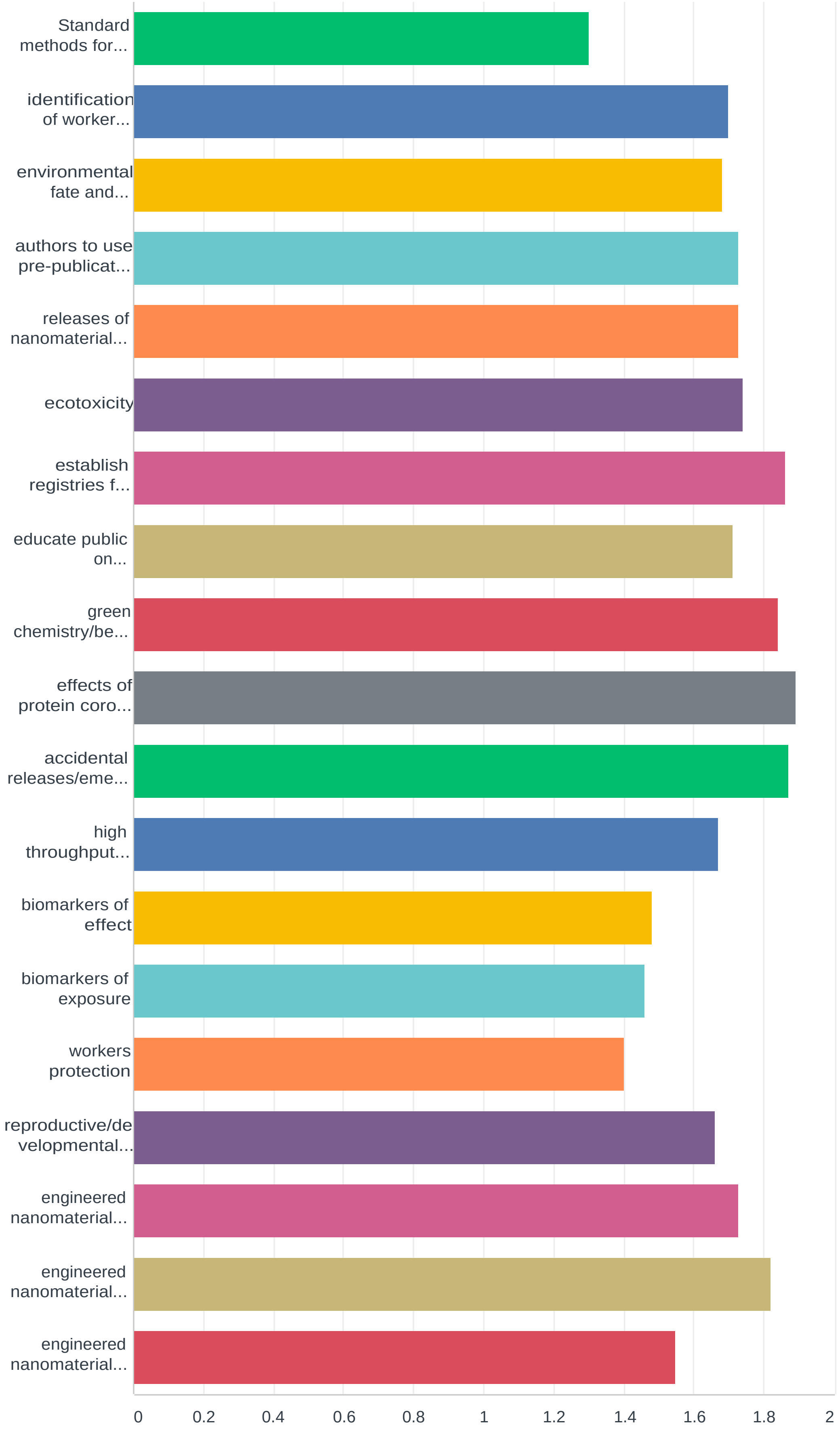
Standard methods for...
identification of worker...
environmental fate and...
authors to use pre-publicat...
releases of nanomaterial...
ecotoxicity
establish registries f...
educate public on...
green chemistry/be...
effects of protein coro...
accidental releases/eme...
high throughput...
biomarkers of effect
biomarkers of exposure
workers protection
reproductive/developmental...
engineered nanomaterial...
engineered nanomaterial...
engineered nanomaterial...
0
0.2
0.4
0.6
0.8
1
1.2
1.4
1.6
1.8
2

	VERY URGENT	SOMEWHAT URGENT	NOT URGENT	TOTAL	WEIGHTED AVERAGE
Standard methods for toxicity testing	71.08%<br>59	27.71%<br>23	1.20%<br>1	83	1.30
identification of worker cohorts to conduct prospective studies	41.67%<br>35	46.43%<br>39	11.90%<br>10	84	1.70
environmental fate and transport	43.21%<br>35	45.68%<br>37	11.11%<br>9	81	1.68
authors to use pre-publication checklist or criteria to publish reproducible results	41.46%<br>34	43.90%<br>36	14.63%<br>12	82	1.73
releases of nanomaterials from aging matrices	38.55%<br>32	49.40%<br>41	12.05%<br>10	83	1.73
ecotoxicity	38.27%<br>31	49.38%<br>40	12.35%<br>10	81	1.74
establish registries for nano workers	34.57%<br>28	44.44%<br>36	20.99%<br>17	81	1.86
educate public on uncertainties/benefits of nanotechnology	41.25%<br>33	46.25%<br>37	12.50%<br>10	80	1.71
green chemistry/benign by design	34.57%<br>28	46.91%<br>38	18.52%<br>15	81	1.84
effects of protein corona on toxic effects	36.59%<br>30	37.80%<br>31	25.61%<br>21	82	1.89
accidental releases/emergency response	25.61%<br>21	62.20%<br>51	12.20%<br>10	82	1.87
high throughput screening tools	48.78%<br>40	35.37%<br>29	15.85%<br>13	82	1.67
biomarkers of effect	59.76%<br>49	32.93%<br>27	7.32%<br>6	82	1.48
biomarkers of exposure	57.83%<br>48	38.55%<br>32	3.61%<br>3	83	1.46
workers protection	64.63%<br>53	30.49%<br>25	4.88%<br>4	82	1.40
reproductive/developmental toxicity	40.00%<br>32	53.75%<br>43	6.25%<br>5	80	1.66
engineered nanomaterials in food products	45.00%<br>36	37.50%<br>30	17.50%<br>14	80	1.73
engineered nanomaterials in environmental media	31.71%<br>26	54.88%<br>45	13.41%<br>11	82	1.82
engineered nanomaterials in human tissues	50.00%<br>41	45.12%<br>37	4.88%<br>4	82	1.55

#	OTHER (PLEASE SPECIFY)	DATE
1	Criteria for grouping and read across, occupational exposure limits, government approval of tools	
2	A lot of more important topics are not included here: research that enables generalization within hazard estimation (omics, AOPs, MIE), predictive estimation of hazards and exposure and "validation", exposure measurement technologies, occupational exposure characteristics and levels, innovation risk governance of NM and NM-enabled products	
3	Disposal of nanomaterials containing products	
4	Immunotoxicity of Nanomaterials	
